# Meiotic nuclear divisions 1 promotes proliferation and metastasis in hepatocellular carcinoma and is a potential diagnostic and therapeutic target gene

**DOI:** 10.1007/s12032-022-01875-w

**Published:** 2022-11-09

**Authors:** Kai Tan, Kunlei Wang, Anbang Zhao, Zhicheng Liu, Wenjing Song, Qian Cheng, Xinyin Li, Zhinan Chen, Yufeng Yuan, Zhiyong Yang

**Affiliations:** 1 Department of Hepatobiliary and Pancreatic Surgery, Zhongnan Hospital of Wuhan University, Wuhan, 430071 Hubei China; 2 Pancreatic Surgery Center, Zhongnan Hospital of Wuhan University, Wuhan, 430071 Hubei China; 3 Clinical Medicine Research Center for Minimally Invasive Procedure of Hepatobiliary & Pancreatic Diseases of Hubei Province, Wuhan, 430071 Hubei China

**Keywords:** MND1, Hepatocellular carcinoma, Biomarker

## Abstract

Hepatocellular carcinoma is the cancer with the highest incidence among liver cancers and how to treat this cancer effectively is still a difficult problem we must face. We selected meiotic nuclear divisions 1 (MND1) as the study object by combining data from The Cancer Genome Atlas (TCGA) database with prognostic survival analysis. We validated the value of MND1 in evaluating the prognosis of hepatocellular carcinoma through a diagnostic and prognostic model. At the same time, cellular experiments were used to demonstrate the effect of MND1 on hepatocellular carcinoma proliferation and migration. We used short hairpin RNA (shRNA) to knock down MND1 in Hun7 and HCCLM3 cell lines. Through 3-(4,5-dimethylthiazol-2-yl)-2,5-diphenyltetrazolium bromide (MTT) and colony formation assays, we found that knocking down MND1 reduced the proliferation of cells. Through wound healing and Transwell assays, we found that knocking down MND1 reduced cell migration and invasion. Moreover, we found that MND1 can promote the proliferation, migration, and invasion of Hep3B cells by overexpressing MND1. Therefore, in general, MND1 is expected to be a gene that can effectively diagnose and treat hepatocellular carcinoma.

**Supplementary Information:**

The online version contains supplementary material available at 10.1007/s12032-022-01875-w.

## Introduction

According to available data, the number of people who die every year because of heart disease ranks first and second cause of death is cancer. In the USA, 690,000 people die yearly from cancer [1]. Hepatocellular carcinoma (HCC) was the third most deadly cancer in the world in 2020 [2]. The extremely high mortality and low cure rate have led us to pay increasing attention to the treatment and prevention of HCC [3]. Although there have been updates and iterations of medical technology in recent years, we still lack an effective method to monitor the early occurrence of cancer [4] and there is no suitable treatment method after diagnosis [5]. In recent decades, molecular targeted therapy has gradually become an important way to treat hepatocellular carcinoma, but the survival efficacy of patients still needs to be improved [6, 7]. Consequently, finding an effective molecular target has become a way to improve the treatment regimen of hepatocellular carcinoma. Therefore, we urgently need to find an effective molecule as a diagnostic marker and therapeutic target for hepatocellular carcinoma [8].

Recently, the popularity of next-generation sequencing technology has made bioinformatics analysis widely used to explore the pathogenesis of various diseases [9, 10]. However, how to use biological analysis to obtain a reliable molecular marker or an effective therapeutic target has always been an urgent problem to be solved.

Meiotic nuclear divisions 1 (MND1) is a protein coding gene considered crucial for meiotic homology repair [11]. Several recent studies have demonstrated that MND1 messenger RNA (mRNA) levels are elevated in lung cancer and breast cancer, which means MND1 mRNA level is considered a prognostic factor for lung cancer and breast cancer [12, 13]. In addition, MND1 has also been proven to promote the cell cycle in lung cancer by activating the KLF6/E2F1-positive feedback loop [14]. However, there are few studies of MND1 in other cancers, especially in hepatocellular carcinoma, and the biological role of MND1 remains unclear.

In this research, we screened more than 100 genes related to the cell cycle through bioinformatics analysis. MND1 is one of the vital hub genes among these genes and is associated with prognosis, so we selected MND1 as our research object. We analyzed the correlation between the mRNA expression level of MND1 and the clinical characteristics of hepatocellular carcinoma patients through an online database. We also conducted in vitro experiments to explore the effect of MND1 knockdown on hepatoma cell lines.

## Materials and methods

### Data collection

We downloaded the mRNA expression levels of MND1 in tumor patients and nontumor patients from GEPI2A (http://gepia.cancer-pku.cn/) [15], and we used The Cancer Genome Atlas (TCGA) database (https://cancergenome.nih.gov) to download the clinical information for those patients. Moreover, MND1 transcript expression levels from the GSE124535, GSE114546, GSE84402, and GSE101685 databases were downloaded to validate our results.

(https://www.ncbi.nlm.nih.gov/geo/).

### Oncomine database analysis

We used the Oncomine database (https://www.oncomine.org/resource/login.html) [16] to obtain the expression levels of MND1 in different cancers. We used Student’s *t* test to judge whether there were differences between cancer tissues and normal tissues. The cutoff *p* value was defined as 0.05.

### Kaplan–Meier plotter analysis

The Kaplan–Meier plotter (http://www.kmplot.com) [17] was used to obtain the overall survival (OS) and relapse-free survival (RFS) of patients. The cutoff *p* value was defined as 0.05.

### LinkedOmics database analysis

The LinkedOmics database (http://www.linkedomics.org) [18] is a multiomics and clinical database of 32 cancer types, as well as data on more than 10,000 patients in TCGA database. Therefore, we downloaded a series of genes coexpressed with MND1 from this database and used volcano plots and heat maps to display them.

### TIMER database analysis

TIMER (http://www.cistrome.shinyapps.io) [19] is a website that can detect immune cell infiltration using RNA-Seq expression profiling data. In this research, TIMER was used to detect the relationship between immune cell-infiltrating cells and MND1 mRNA levels in hepatocellular carcinoma.

### Functional enrichment analysis

To determine the function of differential genes in hepatocellular carcinoma and the pathways involved, we used the “enrichplot” R package for enrichment analysis of Gene Ontology (GO) and Kyoto Encyclopedia of Genes and Genomes (KEGG). The cutoff *p* value was defined as 0.05.

### Cell culture

All liver cancer cell lines (Hun7, HCCLM3, Hep3B, MHCC-97H, HepG2) and HL-7702 (L02) were purchased from the Center for National Collection of Authenticated Cell Cultures (NCACC, China). After testing, there were no mycoplasma, bacteria, or other bacterial contamination. The cultivation methods of Hun7, HCCLM3, MHCC-97H, HepG2, and L02 were in accordance with the instructions on the official website. The cells were cultured in Dulbecco’s modified Eagle’s medium (DMEM) (Gibco, Grand Island, NY, USA) supplemented with 1% penicillin and streptomycin sulfate and 10% fetal bovine serum (FBS; Gibco, USA) and incubated at 37 °C in an incubator containing 5% CO_2_. Hep3B cells were cultured in minimum essential media (MEM) (Gibco, USA) supplemented with 1% penicillin and streptomycin sulfate and 10% fetal bovine serum (FBS; Gibco, USA) and incubated at 37 °C in an incubator containing 5% CO_2_.

### Plasmid construction and transfection

The sequences of shRNA were synthesized chemically and cloned into plasmid PLKO.1. The sequence of MND1 was obtained by polymerase chain reaction (PCR) and cloned into the PCDH plasmid. Then, the hepatoma cell line was stably infected with lentivirus using the transfection reagent Lipor Fiter™ (Let1000, Hanbio, China). The sequences of the shRNAs and primers are listed in Table 1.

### Real-time PCR (RT-PCR)

RNA was extracted using RNA isolater Total RNA Extraction Reagent (R401-01, Vazyme, Nanjing, Jiangsu, China) according to the instructions. All RNAs were then quantified to 1 µg and reverse transcribed into cDNA using HiScript II Q RT SuperMix for quantitative (q)PCR (R222-01, Vazyme, China). We then used ChamQ SYBR qPCR Master Mix (Q311-02, Vazyme, China) to detect gene expression levels and knockdown efficiencies in different cell lines. Primers are listed in Table 1. Gene expression was normalized to the expression of glyceraldehyde-3-phosphate dehydrogenase (GAPDH) and calculated using the 2^−ΔΔCt^ method.

### Western blotting

Extracted protein was lysed on ice for 15 min using radioimmunoprecipitation assay (RIPA) buffer (C510006-0100, Sangon Biotech, Shanghai, China) with protease and phosphatase inhibitors. A bicinchoninic acid (BCA) protein assay kit (C503021-0501, Sangon Biotech, China) was used to detect the protein concentration. Proteins were transferred to polyvinylidene fluoride (PVDF) membranes after being separated by 10% sodium dodecyl sulfate–polyacrylamide gel electrophoresis (SDS-PAGE). Then, the membranes were blocked with 5% skimmed milk at room temperature for one hour. The membranes were incubated overnight with β-actin (1:5000, #8457 Cell Signaling Technology, Danvers, MA, USA), GAPDH (1:5000,10494-1-AP, Proteintech, Wuhan, China) and MND1(1:1000, 11636-1-AP, Proteintech, Wuhan, China) at 4 ℃. The membranes were then incubated with the horseradish peroxidase (HRP)-labeled antibody at room temperature for one hour. Finally, we used electrochemiluminescence (ECL) substrate (hypersensitive) (BL523B, Biosharp, Hangzhou, Zhejiang, China) for exposure.

### MTT assay

All cells were plated in 96-well plates (1000 cells/well). Cell viability was then detected using the MTT Cell Proliferation and Cytotoxicity Assay Kit (E606334, Sangon Biotech, China) according to the instructions. Then, 10 µl of reagent was added to each well, and each well was read with a microplate reader at 570 nm after 4 h of incubation in the cell incubator.

### Colony formation assay

We added approximately 500 cells to each six-well plate and distributed them evenly in each hole by shaking them well. Then, we placed them in an incubator with 5% CO_2_ at 37 °C for 10 days. Each well was washed three times with phosphate-buffered saline (PBS), and the results were analyzed statistically. The cells were then fixed with 4% paraformaldehyde for 15 min and stained with 0.1% crystal violet solution for 20 min.

### Wound healing assay

All cells were plated in 6-well plates. Then, the cells were cultured overnight in fetal bovine serum (FBS)-containing medium. After 24 h, each well was scratched with a 20-µl pipette tip and changed to FBS-free medium. The cells were cultivated and photographed under a microscope at 0, 24, 36, and 48 h. Statistics of cell movement distance used ImageJ.

### Transwell assay

Transparent polyester (PET) Membrane 24-Well 8.0-μM tin (BD Biosciences, Franklin Lakes. NJ, USA) was used to detect the invasive ability of cells. Then, 600 µl of serum-containing medium was added to a 24-well plate, 200 µl of serum-free medium in the upper chamber, and 10,000 cells in the medium. The lower surface was immersed in 4% paraformaldehyde for 20 min, and 0.1% crystal violet solution was used to stain the cells. Five to six mirrors were photographed under the microscope, the number of cells was counted with ImageJ, and the average was taken.

### Statistical analysis

Data are presented as the mean ± standard error of the mean (SEM). Paired Student’s *t* test was performed to assess differences. A *p* value < 0.05 was considered statistically significant.

## Results

### Identification of key genes and pathways in hepatocellular carcinoma by bioinformatics analysis

To determine the genes that play a key role in the occurrence and development of hepatocellular carcinoma, we used bioinformatics analysis to analyze the differentially expressed genes in GSE101432, including 719 upregulated differentially expressed genes (DEGs) and 1062 downregulated DEGs. We made a heatmap (Fig. S1A) with the top 200 DEGs and a volcano map (Fig. S1B) with the DEGs. As shown in Fig. S1C, enrichment analysis revealed that the DEGs almost mapped to the DNA replication GO terms. KEGG enrichment analysis also displayed the enrichment of DNA replication (Fig. S1D). These data suggest that the cell cycle plays a critical role in the progression of hepatocellular carcinoma. Therefore, we took an intersection of upregulated DEGs of GSE101432, GSE84402, GSE101685, and cell cycle-related genes from GeneCards. As shown in Fig. S1E, there are 101 DEGs. MND1 is one of the key hub genes among these genes and is associated with prognosis, so we further explored its role in hepatocellular carcinoma.

### MND1 is overexpressed in hepatocellular carcinoma

**Fig. 1:**
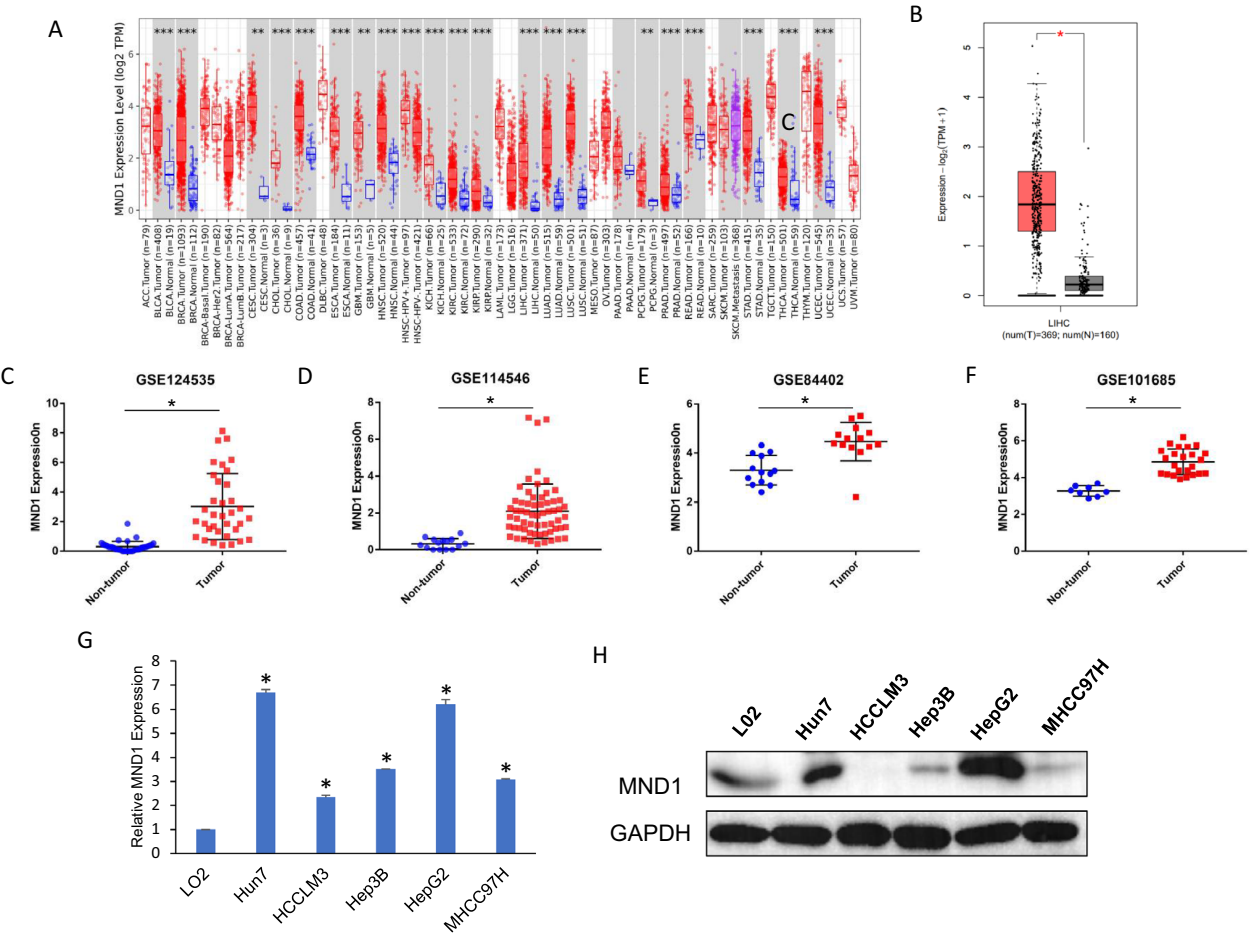
MND1 was overexpressed in Hepatocellular Carcinoma. **A** Expression of MND1 in different cancers. **B** MND1 mRNA expression levels in hepatocellular carcinoma tissues and normal tissues. **C**–**F** Expression of MND1 in tumor and unpaired tissues of the GSE124535, GSE114546, GSE84402, and GSE1016859 datasets in the Gene Expression Omnibus (GEO) data. **G** qPCR detecting the mRNA expression level of MND1 in different hepatocellular carcinoma cell lines. **H** Western blot detecting the protein expression level of MND1 in different hepatocellular carcinoma cell lines. *p < 0.05

To explore the expression of MND1 in tumor and nontumor tissues, we used the Oncomine database to analyze the expression of MND1 in different cancers. The results are shown in Fig. 1A. Compared with normal tissues, the expression of MND1 was highly increased in 21 types of cancer, including hepatocellular carcinoma (LIHC). At the same time, we also verified the expression of MND1 in hepatocellular carcinoma with the online database GEPI2A, which also showed high expression (Fig. 1B). Furthermore, we analyzed MND1 expression in HCC and adjacent nontumor tissues using four other datasets downloaded from GSE124535, GSE114546, GSE84402, and GSE101685, which showed that MND1 mRNA expression was increased in HCC compared with adjacent tissues (Fig. 1C–F). Furthermore, we used Western blotting and qPCR to explore MND1 expression in different hepatoma cell lines. As shown in Fig. 1G–H, MND1 expression was higher than the MND1 expression in the immortalized liver cell Line L02.

### MND1 expression is associated with clinicopathological features and survival

**Fig. 2:**
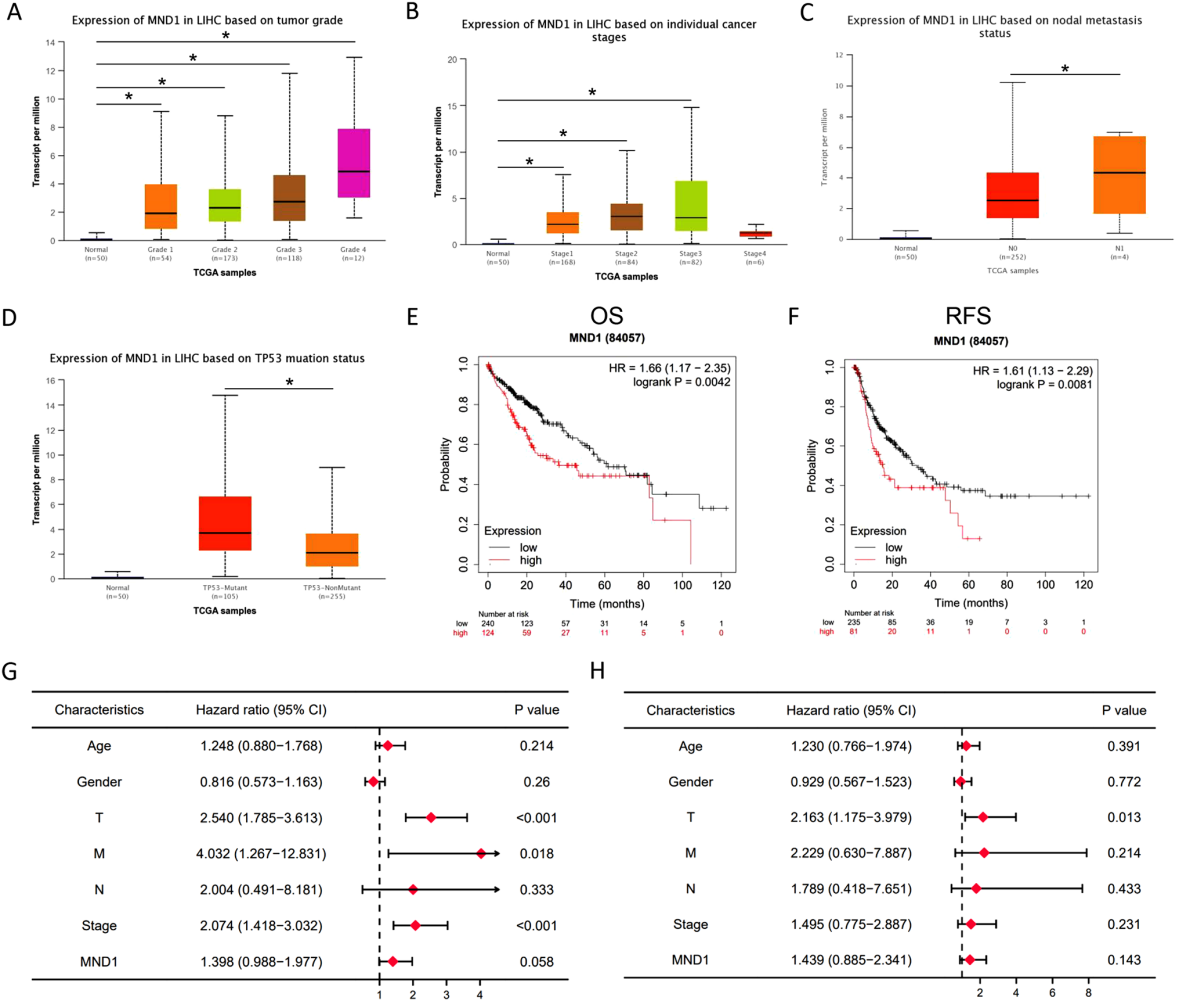
MND1 expression is associated with clinicopathological features and survival. **A** MND1 expression level is correlated with tumor grade. **B** MND1 expression level is correlated with individual cancer stages. **C** MND1 expression level is correlated with nodal metastasis. **D** MND1 expression level is correlated with TP53 mutation status. **E**–**F** Overall and relapse-free survival curves of HCC patients. **G** Tree diagram of a univariate regression analysis. **H** Tree diagram of a multivariate regression analysis. *p < 0.05

To explore the correlation between MND1 and clinicopathological features, we downloaded clinical information from TCGA (Table 2). The results show that the expression of MND1 is associated with various clinical characteristics of patients. Furthermore, we used the UALCAN database to explore the relationship between MND1 and the pathological features of hepatocellular carcinoma patients. As shown in Fig. 2A–D, the expression of MND1 was highly correlated with tumor grade, individual cancer stage, nodal metastasis, and TP53 mutation status. Kaplan–Meier survival analysis is shown in Fig. 2E–F. MND1 expression was correlated with the overall and relapse-free survival curves of HCC patients. Furthermore, we also performed univariate and multivariate analyses. Univariate analysis (Fig. 2G) indicated that high MND1 expression T stage, N stage, and TNM stage predicted worse overall survival time. The multivariate analysis showed (Fig. 2H) that only the T stage was independently associated with the overall survival time. Overall, high levels of MND1 may be associated with tumor stage and worse survival.

### Diagnostic value of MND1 expression in LIHC

**Fig. 3:**
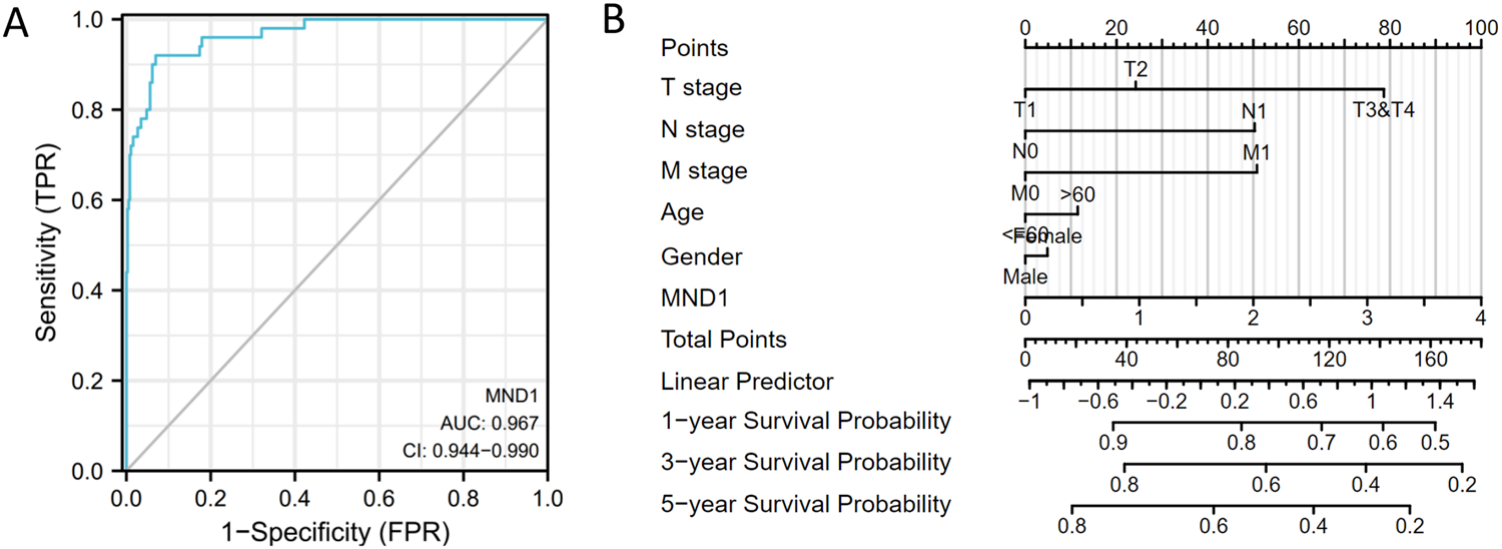
Diagnostic value of MND1 expression in LIHC. **A** Receiver operating characteristic (ROC) curve analysis for MND1 expression in LIHC and adjacent tissue. **B** Nomogram survival prediction chart for predicting the 1-, 3-, and 5-year overall survival rates

To determine the diagnostic value of MND1 in hepatocellular carcinoma, we constructed a receiver operating characteristic (ROC) plot using TCGA database. As shown in the plot in Fig. 3A, the area under the ROC curve (AUC) was 0.967, suggesting that MND1 is of great significance in diagnosing hepatocellular carcinoma. In addition, we combined the expression levels of mnd1 with clinical variables to construct a nomogram to predict the 1-, 3-, and 5-year survival probabilities of patients (Fig. 3B).

### Coexpression genes correlated with MND1 in hepatocellular carcinoma

**Fig. 4:**
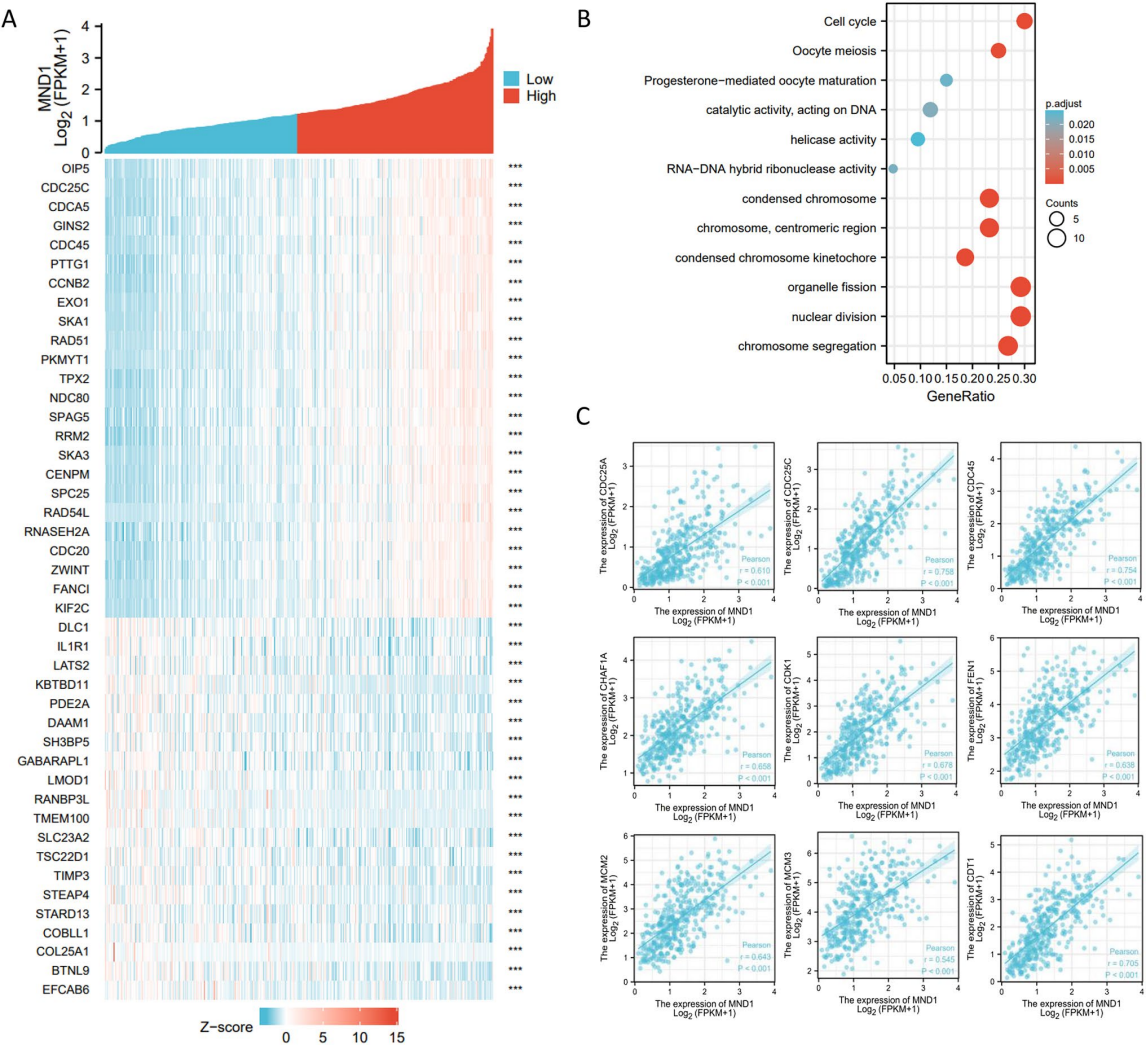
The coexpression gene of MND1 predicts that MND1 may be related to cell cycle. **A** Heatmap showing the top 50 genes in hepatocellular carcinoma (LIHC) that were positively and negatively related to MND1. Red represents positively related genes and blue represents negatively related genes. **B** Gene Ontology (GO) term and Kyoto Encyclopedia of Genes and Genomes (KEGG) pathway analyses of MND1-related genes in LIHC. **C** Pearson correlation analysis of MND1 and cell cycle-related genes (CDC25A, CDC25C, CDC25, CHAF1A, CDK1, FEN1, MCM2, MCM3, CDT1) in TCGA-Liver Hepatocellular Carcinoma (LIHC) dataset

To explore genes coexpressed with MND1 in hepatocellular carcinoma, we performed coexpression analysis using LinkedOmics. The volcano map shown in Fig. S2A shows a series of genes positively and negatively associated with MND1. Fig. S2B shows 50 genes positively correlated with MND1, and Fig. S2C shows 50 genes negatively correlated with MND1. Furthermore, to explore the biological function of MND1 in hepatocellular carcinoma, we made a coexpression heatmap using the top 25 positively and negatively correlated genes among the coexpressed genes (Fig. 4A), and we made an enriched GO functional enrichment and KEGG pathways among the MND1-related genes. As shown in Fig. 4B, these genes are suggested to be significantly associated with biological activities, such as the cell cycle and cell division, which means that MND1 is one of the crucial genes in tumor progression. We further analyzed the correlation between MND1 and cell cycle-related factors, and the results suggested that there was a significant relationship between MND1 and CDC25A, CDC25C, CDC45, CHAF1A, CDK1, FEN1, MCM2, MCM3, and CDT1, which further proved that MND1 is a crucial gene associated with tumor progression (Fig. 4C).

### Correlation analysis of MND1 expression and immune infiltration in hepatocellular carcinoma

Finally, we also explored the relationship between MND1 and immune infiltration based on the Spearman correlation coefficient. As shown in Fig. S3A, MND1 expression was negatively correlated with the abundance of neutrophils, eosinophils, mast cells, CD8 T cells, Th17 cells, Tcm cells, DCs, Tgd Tem cells, iDCs, and natural killer (NK) cells and was positively correlated with the abundance of Th2 cells, TFH cells, and aDCs. In addition, we used the TIMER database to explore the relationship between MND1 transcription and immune cell infiltration (Fig. S3B). We found that the expression level of MND1 and multiple immune cells showed a positive correlation.

### Knockdown of MND1 inhibited HCC proliferation, cell invasion, and cell migration in vitro

**Fig. 5:**
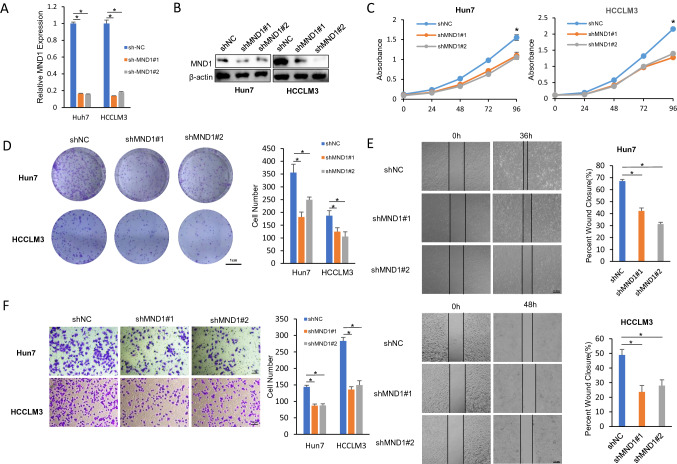
Knockdown MND1 inhibited HCC proliferation, cell invasion, and cell migration in vitro. **A**–**B** MND1 knockdown efficiency were confirmed by qPCR and Western blotting in Huh7 and HCCLM3 cell. **C** MTT assay was used to detect cell viability in Hun7 and HCCLM3 cells. **D** Colony formation assay was used to detect cell proliferation in Hun7 and HCCLM3 cells. **E** Wound healing assay was used to detect cell migration in Hun7 and HCCLM3 cells. **F** Transwell assay was used to detect cell invasion in Hun7 and HCCLM3 cells. *p < 0.05

To further explore the role of MND1 in hepatocellular carcinoma, we performed in vitro experiments. First, we constructed two pairs of shRNAs and made them stably expressed in Hun7 and HCCLM3 cells by lentivirus. We used qPCR and Western blotting to detect their expression in cells. The results (Fig. 5A–B) suggest that they were knocked down. The MTT assay and colony formation assay results (Fig. 5C–D) showed that knockdown of MND1 inhibited cell proliferation. In addition, we performed a cell scratch test and Transwell test to detect the migration and invasion abilities of cells. As shown in Fig. 5E and F, knockdown of MND1 inhibited cell migration and invasion. Overall, knockdown of MND1 in Hun7 and HCCLM3 cells inhibited growth, migration, and invasion.

### MND1 overexpression promoted cell proliferation, migration, and invasion

**Fig. 6:**
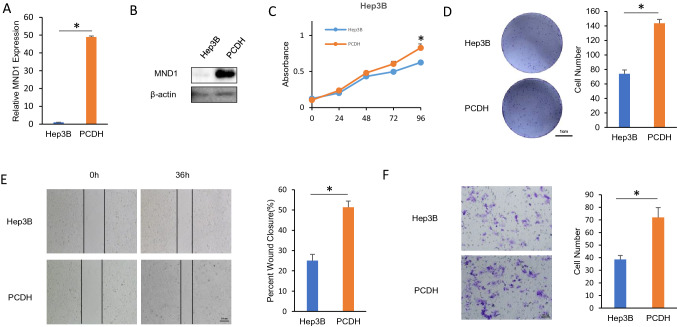
MND1 overexpression promoted cell proliferation, migration, and invasion of Hep3B. **A**–**B** MND1 overexpression efficiency was confirmed by qPCR and Western blotting in Hep3B cells. **C** MTT assay was used to detect cell viability in Hep3B cells. **D** Colony formation assay was used to detect cell proliferation in Hep3B cells. **E** Wound healing assay was used to detect cell migration in Hep3B cells. **F** Transwell assay was used to detect cell invasion in Hep3B cells. *p < 0.05

Since knockdown of MND1 promotes the progression of liver cancer cell lines, we wondered whether overexpression of MND1 in liver cancer cell lines would promote its progression. Therefore, we constructed an MND1-overexpressing cell line in Hep3B cells. As shown in Fig. 6A and B, the expression of MND1 in PCDH cells was highly increased compared with the expression of MND1 in Hep3B cells. As we previously speculated, overexpression of MND1 promoted cell proliferation, migration, and invasion (Fig. 6C–F).

## Discussion

Hepatocellular carcinoma is one of the top five cancers in the world in terms of cancer prevalence [2]. A correct understanding of the mechanism of the occurrence and development of hepatocellular carcinoma is crucial to correctly understand the disease and find effective treatment amplification [20–22]. Bioinformatics is an emerging discipline that can be applied to reveal the molecular mechanisms of gene expression regulation in model organisms [23]. In our study, we first downloaded the GSE101432 dataset from Gene Expression Omnibus (GEO) data and obtained a series of upregulated and downregulated genes through bioinformatics analysis. We performed KEGG and GO enrichment analyses on these differentially expressed genes. The most significant pathways found to be enriched were during the cell cycle and cell division. As many previous studies reported, one of cancer’s most important manifestations is its strong proliferative capacity [24–26]. Therefore, we downloaded multiple datasets from the GEO dataset again to analyze the upregulated and downregulated genes, and at the same time, we downloaded the genes significantly related to the cell cycle from the GeneCard database and performed an intersection between them. In the end, 101 genes were obtained. MND1 is a gene that plays a vital role in meiosis [27–30], but its relationship with cancer is unclear. Although it has been reported to be associated with lung cancer, breast cancer, and renal clear cell carcinoma [12, 13, 31], the relationship between MND1 and cancer has not been well explained [32]. Ultimately, we selected one MND1 as our study subject.

Previous studies suggest that MND1 may be a powerful target for tumor therapy [33–36]. In our study, we analyzed the expression of MND1 in various cancers. The results showed that MND1 was highly expressed in many cancers. As previously reported, MND1 was highly expressed in both lung cancer and adenocarcinoma [12, 13]. We also found that MND1 is highly expressed in hepatocellular carcinoma and verified the high expression of MND1 in hepatocellular carcinoma cell lines. These results suggested that MND1 may play a role in promoting the occurrence and development of hepatocellular carcinoma. In this study, we analyzed the role of MND1 in prognostic diagnosis through TCGA database and demonstrated that MND1 has prognostic value in hepatocellular carcinoma. Furthermore, we also analyzed genes coexpressed with MND1 and demonstrated a strong association between MND1 and the cell cycle and proliferation. The cell cycle is an important system that can regulate cell proliferation and division [37]. As a previous study showed, CDK1 is the most important cell cycle CDK and is required for the successful completion of M-phase. CDC25A and CDC25C are crucial for the G2/M transition [38]. MCM2, MCM3, and CDC45 are essential for the early steps of DNA replication [39, 40]. There are also many other genes that regulate cell proliferation, such as FEN1, CHAF1A, and CDT1 [41–43]. We found that these genes are correlated, which further proves our conclusion. One study reported an association between the high expression of MND1 and the immune microenvironment in breast cancer [13]. Similar to previous results, we also obtained consistent results in hepatocellular carcinoma.

A study has previously demonstrated that MND1 can promote cell proliferation and migration in lung cancer and knockdown inhibits the proliferation and migration of lung cancer cells [14]. However, no other studies have demonstrated the role of MND1 in other cancers. In our research, by knocking down MND1 in liver cancer cell lines, we confirmed that knockdown could affect the phenotypes of hepatocellular carcinoma, such as proliferation and migration. We also performed overexpression experiments to prove that MND1 promotes the proliferation and migration of hepatocellular carcinoma. These in vitro experiments demonstrate that knockdown of MND1 in hepatocellular carcinoma likely alleviates hepatocellular carcinoma progression by inhibiting proliferation and migration. However, this study has certain limitations, and while we validated it in vitro, we did not validate it in vivo. At the same time, the mechanism by which MND1 leads to the progression of hepatocellular carcinoma remains to be further studied, and we will conduct more in-depth research on this topic in future.

## Conclusions

In the present study, we analyzed the diagnostic value of MND1 in hepatocellular carcinoma using TCGA database and verified by in vitro experiments that MND1 may promote the progression of hepatocellular carcinoma by promoting proliferation and migration. MND1 is expected to become a new diagnostic marker and an effective therapeutic target.

**Table 1:** Primer sequences and shRNA used in this study

Primer name	Sequence (5′–3′)
GAPDH	F: GTCTCCTCTGACTTCAACAGCG R: ACCACCCTGTTGCTGTAGCCAA
MND1	F: TGTGAGAGGATCGGAACTTCT R: CACATCGGCCAATTTTAGCTTTC
shNC	TTTCCCGAACGTGTCACGT
shMND1#1	GCATTACTGCTATGTCAGTAA
shMND1#2	GCTGCTAACAGATGGACTGAT

**Table 2:** Correlation between clinicopathological features and MND1 expression in HCC tumor tissues

Characteristic	Low expression of MND1	High expression of MND1	p
n	185	186	
T stage, *n* (%)			0.002
T1	107 (29.1%)	74 (20.1%)	
T2	38 (10.3%)	56 (15.2%)	
T3	34 (9.2%)	46 (12.5%)	
T4	3 (0.8%)	10 (2.7%)	
N stage, * n* (%)			1.000
N0	122 (47.7%)	130 (50.8%)	
N1	2 (0.8%)	2 (0.8%)	
M stage, * n* (%)			0.358
M0	128 (47.4%)	138 (51.1%)	
M1	3 (1.1%)	1 (0.4%)	
Pathologic stage, * n* (%)			0.001
Stage I	101 (29.1%)	70 (20.2%)	
Stage II	33 (9.5%)	53 (15.3%)	
Stage III	34 (9.8%)	51 (14.7%)	
Stage IV	4 (1.2%)	1 (0.3%)	
Gender, * n* (%)			0.853
Female	59 (15.9%)	62 (16.7%)	
Male	126 (34%)	124 (33.4%)	
Age, * n* (%)			0.532
≤ 60	85 (23%)	92 (24.9%)	
> 60	100 (27%)	93 (25.1%)	
Residual tumor, * n* (%)			0.465
R0	165 (48.2%)	159 (46.5%)	
R1	7 (2%)	10 (2.9%)	
R2	1 (0.3%)	0 (0%)	
Vascular invasion, * n* (%)			1.000
No	107 (34%)	99 (31.4%)	
Yes	56 (17.8%)	53 (16.8%)	
Age, median (IQR)	62 (52, 69)	61 (51, 68)	0.262

## Supplementary Information

Below is the link to the electronic supplementary material.

Supplementary file1 (DOCX 13 kb)

Supplementary file2 (PDF 752 kb)

## Data Availability

The datasets used for the current study are available from the corresponding authors upon reasonable request.

## References

[1] Siegel RL, Miller KD, Fuchs HE (2022). Cancer statistics, 2022. CA Cancer J Clin.

[2] Sung H, Ferlay J, Siegel RL (2021). Global Cancer Statistics 2020: GLOBOCAN estimates of incidence and mortality worldwide for 36 cancers in 185 countries. CA Cancer J Clin.

[3] Vogel A, Cervantes A, Chau I (2018). Hepatocellular carcinoma: ESMO Clinical Practice Guidelines for diagnosis, treatment and follow-up. Ann Oncol.

[4] Zhang CH, Cheng Y, Zhang S (2022). Changing epidemiology of hepatocellular carcinoma in Asia. Liver Int.

[5] Heimbach JK, Kulik LM, Finn RS (2018). AASLD guidelines for the treatment of hepatocellular carcinoma. Hepatology.

[6] Lee YT, Tan YJ, Oon CE (2018). Molecular targeted therapy: treating cancer with specificity. Eur J Pharmacol.

[7] Khan AA, Liu ZK, Xu X (2021). Recent advances in immunotherapy for hepatocellular carcinoma. Hepatobiliary Pancreat Dis Int.

[8] Robb MA, McInnes PM, Califf RM (2016). Biomarkers and surrogate endpoints: developing common terminology and definitions. JAMA.

[9] Huo J, Cai J, Wu L (2022). Comprehensive analysis of metabolic pathway activity subtypes derived prognostic signature in hepatocellular carcinoma. Cancer Med.

[10] Tao Z, Shi A, Li R (2017). Microarray bioinformatics in cancer—a review. J Buon.

[11] Kang HA, Shin HC, Kalantzi AS (2015). Crystal structure of Hop2-Mnd1 and mechanistic insights into its role in meiotic recombination. Nucleic Acids Res.

[12] Wei J, Meng G, Wu J (2021). Genetic network and gene set enrichment analyses identify MND1 as potential diagnostic and therapeutic target gene for lung adenocarcinoma. Sci Rep.

[13] Bao Z, Cheng J, Zhu J (2022). Using weighted gene co-expression network analysis to identify increased MND1 expression as a predictor of poor breast cancer survival. Int J Gen Med.

[14] Zhang Q, Shi R, Bai Y (2021). Meiotic nuclear divisions 1 (MND1) fuels cell cycle progression by activating a KLF6/E2F1 positive feedback loop in lung adenocarcinoma. Cancer Commun (Lond).

[15] Tang Z, Kang B, Li C (2019). GEPIA2: an enhanced web server for large-scale expression profiling and interactive analysis. Nucleic Acids Res.

[16] Rhodes DR, Kalyana-Sundaram S, Mahavisno V (2007). Oncomine 3.0: genes, pathways, and networks in a collection of 18,000 cancer gene expression profiles. Neoplasia.

[17] Lanczky A, Gyorffy B (2021). Web-based survival analysis tool tailored for medical research (KMplot): development and implementation. J Med Internet Res.

[18] Vasaikar SV, Straub P, Wang J (2018). LinkedOmics: analyzing multi-omics data within and across 32 cancer types. Nucleic Acids Res.

[19] Li T, Fu J, Zeng Z (2020). TIMER2.0 for analysis of tumor-infiltrating immune cells. Nucleic Acids Res.

[20] Jiang Y, Han QJ, Zhang J (2019). Hepatocellular carcinoma: mechanisms of progression and immunotherapy. World J Gastroenterol.

[21] Alqahtani A, Khan Z, Alloghbi A (2019). Hepatocellular carcinoma: molecular mechanisms and targeted therapies. Medicina (Kaunas).

[22] Marengo A, Rosso C, Bugianesi E (2016). Liver cancer: connections with obesity, fatty liver, and cirrhosis. Annu Rev Med.

[23] Orlov YL, Tatarinova TV, Anashkina AA (2021). Bioinformatics applications to reveal molecular mechanisms of gene expression regulation in model organisms. Int J Mol Sci.

[24] Hanahan D (2022). Hallmarks of cancer: new dimensions. Cancer Discov.

[25] Nagarajan S, Rao SV, Sutton J (2020). ARID1A influences HDAC1/BRD4 activity, intrinsic proliferative capacity and breast cancer treatment response. Nat Genet.

[26] Aragona M, Panciera T, Manfrin A (2013). A mechanical checkpoint controls multicellular growth through YAP/TAZ regulation by actin-processing factors. Cell.

[27] Pezza RJ, Voloshin ON, Vanevski F (2007). Hop2/Mnd1 acts on two critical steps in Dmc1-promoted homologous pairing. Genes Dev.

[28] Bugreev DV, Huang F, Mazina OM (2014). HOP2-MND1 modulates RAD51 binding to nucleotides and DNA. Nat Commun.

[29] Petukhova GV, Pezza RJ, Vanevski F (2005). The Hop2 and Mnd1 proteins act in concert with Rad51 and Dmc1 in meiotic recombination. Nat Struct Mol Biol.

[30] Zierhut C, Berlinger M, Rupp C (2004). Mnd1 is required for meiotic interhomolog repair. Curr Biol.

[31] Fang J, Zhen J, Gong Y (2022). MND1 functions as a potential prognostic biomarker associated with cell cycle and immune infiltration in kidney renal clear cell carcinoma. Aging (Albany NY).

[32] Dastsooz H, Cereda M, Donna D (2019). A comprehensive bioinformatics analysis of UBE2C in cancers. Int J Mol Sci.

[33] McFarlane RJ, Wakeman JA (2017). Meiosis-like functions in oncogenesis: a new view of cancer. Cancer Res.

[34] Simpson AJ, Caballero OL, Jungbluth A (2005). Cancer/testis antigens, gametogenesis and cancer. Nat Rev Cancer.

[35] Whitehurst AW (2014). Cause and consequence of cancer/testis antigen activation in cancer. Annu Rev Pharmacol Toxicol.

[36] Fratta E, Coral S, Covre A (2011). The biology of cancer testis antigens: putative function, regulation and therapeutic potential. Mol Oncol.

[37] Coffman JA (2004). Cell cycle development. Dev Cell.

[38] Bouldin CM, Kimelman D (2014). Cdc25 and the importance of G2 control: insights from developmental biology. Cell Cycle.

[39] Ishimi Y (2018). Regulation of MCM2-7 function. Genes Genet Syst.

[40] Aparicio T, Ibarra A, Mendez J (2006). Cdc45-MCM-GINS, a new power player for DNA replication. Cell Div.

[41] Zheng L, Jia J, Finger LD (2011). Functional regulation of FEN1 nuclease and its link to cancer. Nucleic Acids Res.

[42] Tao L, Moreno-Smith M, Ibarra-Garcia-Padilla R (2021). CHAF1A blocks neuronal differentiation and promotes neuroblastoma oncogenesis via metabolic reprogramming. Adv Sci (Weinh).

[43] Rialland M, Sola F, Santocanale C (2002). Essential role of human CDT1 in DNA replication and chromatin licensing. J Cell Sci.

