# Supplementary material for: Meiotic nuclear divisions 1 promotes proliferation and metastasis in hepatocellular carcinoma and is a potential diagnostic and therapeutic target gene
Source: Med Oncol. 2022 Nov 9;40(1):14. doi: 10.1007/s12032-022-01875-w (PMC9646579; doi:10.1007/s12032-022-01875-w)
Supplement: Supplementary file 1 — Supplementary file1 (DOCX 13 kb) [file 12032_2022_1875_MOESM1_ESM.docx]

Figure S1. Identification of Key Genes and Pathways in Hepatocellular Carcinoma by Bioinformatics Analysis useing GSE 101432.

(A) Heatmap of the GSE101432 dataset. (B) Volcano plot. (C) GO pathway enrichment analysis (BP, biological process; CC, cell component; MF, molecular function). (D) KEGG analysis. (E) Joint analysis of multiple datasets predicts MND1 as hub gene.

Figure S2.Genes differentially expressed in correlation with MND1 in hepatocellular carcinoma (LinkedOmics).

(A) Volcano map showing genes coexpressed with MND1 in LIHC. (B-C) Heatmaps showing genes positively and negatively correlated with MND1 in LIHC (TOP 50). Red indicates positively correlated genes, and green indicates negatively correlated genes.

Figure S3. Correlation analysis of MND1 expression and immune infiltration in hepatocellular carcinoma.

(A) Correlations between the relative abundance of 24 immune cells and MND1 expression levels. (B) Assessment of the relationship between MND1 expression and six-type immune cell score from TIMER database.
