# Supplementary figures and images for: Meiotic nuclear divisions 1 promotes proliferation and metastasis in hepatocellular carcinoma and is a potential diagnostic and therapeutic target gene
Source: Med Oncol. 2022 Nov 9;40(1):14. doi: 10.1007/s12032-022-01875-w (PMC9646579; doi:10.1007/s12032-022-01875-w)

Figure S1

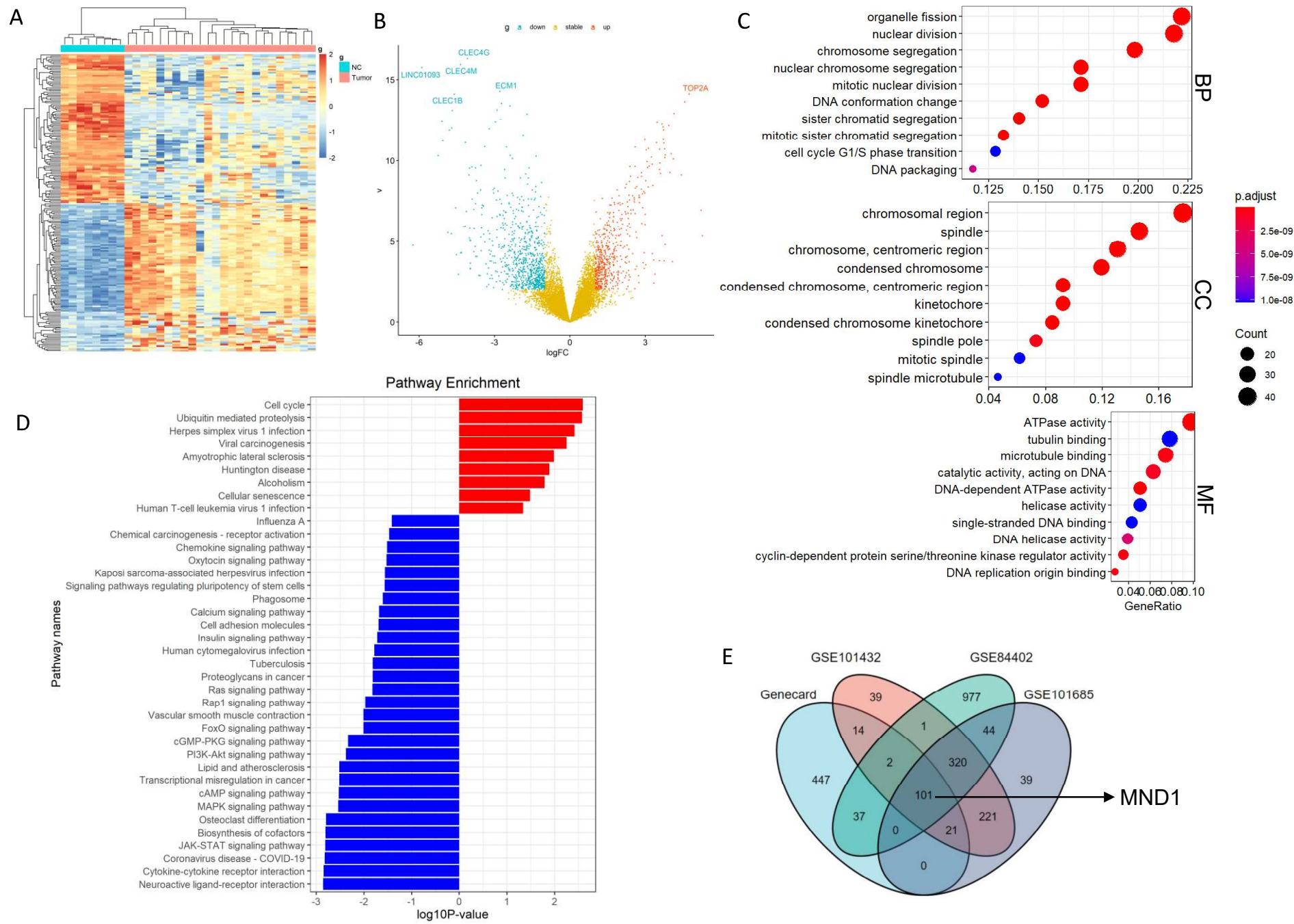

Figure S2

A

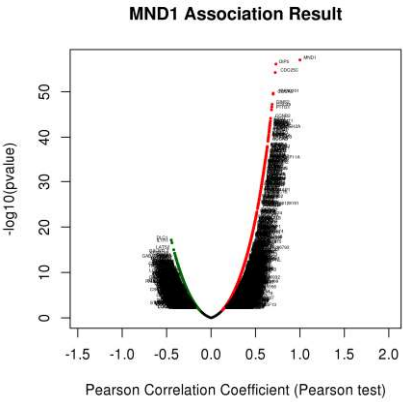

B

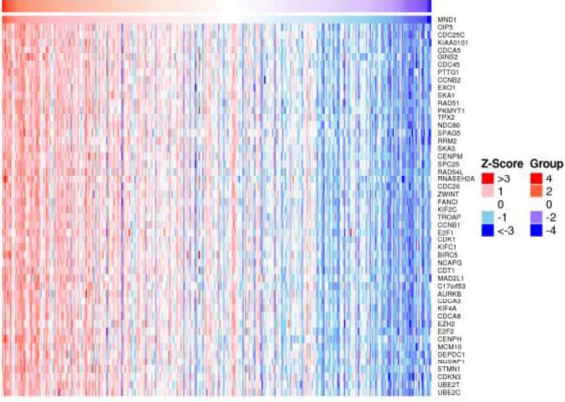

C

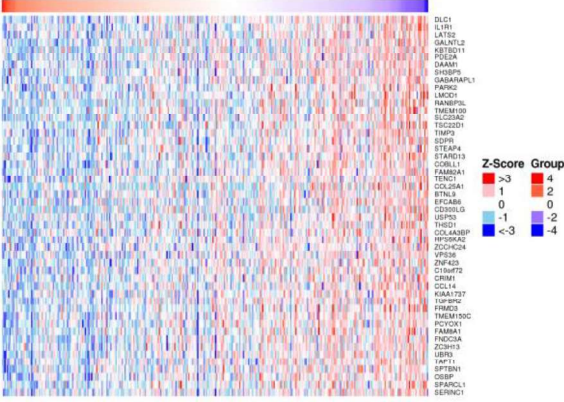

Figure S3

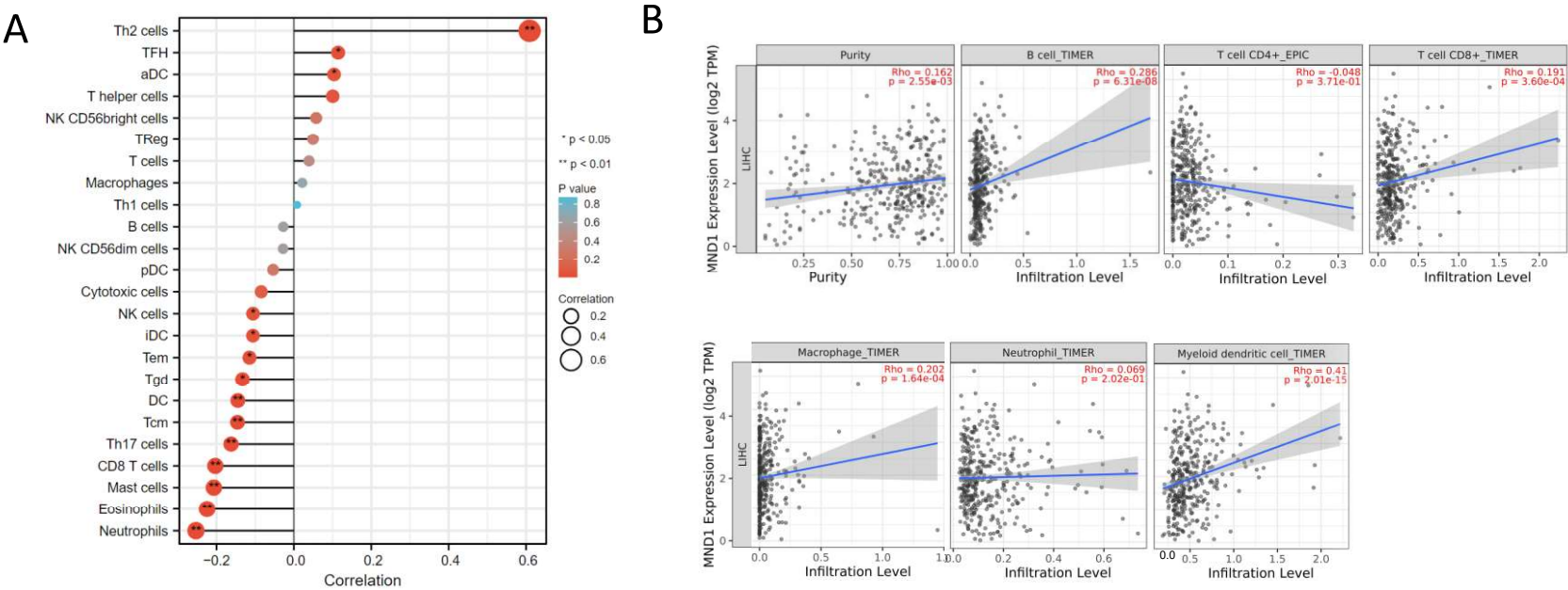

Supplement: Supplementary file 2 — Supplementary file2 (PDF 752 kb) [file 12032_2022_1875_MOESM2_ESM.pdf]
